# Correlation between Ocular and Rectal Temperature with Intra Ocular Pressure in Horse during Exercise

**DOI:** 10.3390/ani12141850

**Published:** 2022-07-21

**Authors:** Francesca Aragona, Simona Di Pietro, Francesca Arfuso, Francesco Fazio, Giuseppe Piccione, Elisabetta Giudice, Claudia Giannetto

**Affiliations:** Department of Veterinary Sciences, University of Messina, 98168 Messina, Italy; fraragona@unime.it (F.A.); farfuso@unime.it (F.A.); ffazio@unime.it (F.F.); gpiccione@unime.it (G.P.); egiudice@unime.it (E.G.); clgiannetto@unime.it (C.G.)

**Keywords:** rectal temperature, ocular temperature, intraocular pressure, exercise, horses

## Abstract

**Simple Summary:**

The athletic horse has been found to be sensitive to numerous metabolic and physiological changes during physical exercise, making it a stressor which may go on to affect thermal and homeostatic well-being. In the present study we aimed to evaluate some ocular physiological variables (ocular temperature and intraocular pressure) and how they may be correlated to the rectal temperature, which better describes the thermoregulatory system. This study was conducted on 14 healthy horses from the same horse training center. Ocular temperature, intraocular pressure and rectal temperature were evaluated under different experimental conditions (*am* and *pm* hours) and before and after a show jumping exercise protocol. Ocular and rectal temperature increased after the exercise as a result of muscle metabolism activation, increasing blood flow in several regions of the body to improve oxygen supply and heat dissipation. On the contrary, intraocular pressure significantly decreased after exercise, probably due to peripheral vasodilatation. Our results showed a correlation between increased body temperatures and IOP variations during exercise. It would be interesting to consider these variables as indicators to determine physiological status of horses during physical exercise.

**Abstract:**

The aim of the present study was to investigate the response to physical exercise of some ocular physiological variables (ocular temperature and intraocular pressure) in horses performing a jumping course in the morning (a.m.) or in the afternoon hours (p.m.), before and after the exercise, in correlation with the rectal temperature. Data collection was carried out on 14 clinical healthy Italian saddle horses. All horses were trained from 9:00 to 10:00 a.m. and from 19:00 to 20:00 p.m. according to a specific training program. Ocular temperature (OT), rectal temperature (RT) and intraocular pressure (IOP) were determined. Statistical analysis showed no differences between the results for the left and the right eyes. The application of two-way repeated-measures analysis of variance (ANOVA) showed a statistically significant effect of time (before vs. after) on IOP (*p* = 0.0001). RT and OT were statistically influenced by time of day and by experimental conditions (a.m. vs. p.m.) (*p* = 0.0001). Bland–Altman (B-A) testing showed an agreement between the values of RT and OT. Our results showed a correlation between increased body temperatures and IOP variations during exercise, so they can probably be considered indicators of athletic horses’ physical fitness during exercise.

## 1. Introduction

Physical exercise is considered a natural stressor situation that produces biological responses, such as the alteration of blood flow and heat loss, by affecting horses’ thermal welfare and homeostasis [1]. During physical exercise, a horse’s metabolic rate increases to transport oxygen, water electrolytes, nutrients and hormones to the contracting muscles by elevating blood pressure, the heart rate and internal body temperature by activating thermoregulatory mechanisms to dissipate excess heat, as hyperthermia induced by physical exercise may be hazardous to health and physical performance [2,3]. The relationship between stress and the metabolic system has been well documented in mammals using various invasive methods, such as blood sampling, the measurement of cortisol in saliva, thermal microchips, tympanic infrared recording and body temperature measurements [4]. Body temperature is considered an important indicator of welfare in animals as well as in humans, and has been used to monitor clinical status in assessments of fever, stress or infectious animal disease [5,6].

Ocular temperature (OT), which is easily measurable, is used as a physiological parameter as it may reflect body temperature changes in response to events and stimuli, and this is accomplished by means of infrared technology (IRT). As it reflects body temperature, the measurement of OT can be used to monitor acute and chronic stress levels in horses during physical exercise and competitions [7,8,9,10,11].

Recently, an innovative method of body temperature monitoring has been introduced. Infrared thermography (IRT) is a passive, rapid, non-invasive method, easily accepted by the animals, which is used to quantify body surface temperature. IRT uses a specialized camera, similar to a digital camera, that measures surface temperature and thermal radiation emitted from an object, displaying the different temperatures detected as an image, with different temperatures denoted by different colors and shades. In order for an IRT system to be used to measure body temperature, the surface location needs to correlate with the changes in body temperature; one possible location is the ocular region [12].

Intraocular pressure (IOP) is likewise considered a fundamental parameter of ocular health and disease and it is used as part of routine investigations in ocular examinations [13,14,15]. It is used to ensure the diagnosis and management of glaucoma in humans and domestic animals when elevated levels of IOP appear, whereas lower values are indicative of anterior uveitis and the post-operative management of corneal, lenticular and vitreoretinal diseases [16,17,18,19,20].

IOP values are regulated by a continuous secretion of aqueous humor from the ciliary epithelium that drains from the eye through the canal of Schlemm and the uveoscleral pathway [15]. There are many controversial opinions about the relationship between physical exercise and decreases in IOP after exercise [3,21]. Some authors have reported that the IOP reduction is directly related to the exercise load [22,23,24]. Numerous studies have shown that daily exercise reduces intraocular pressure (IOP) as it is positively correlated with systemic blood pressure, promoting hypotension. For this reason, sports are thus considered essential for preventing common ocular diseases or yielding some benefit for patients with glaucoma and high ocular tension [25,26].

IOP, OT and RT in particular have been monitored during both the a.m. and p.m. hours as this reflects the impact of endogenous circadian rhythms, in addition to workload conditions or environmental changes. Knowledge about time-of-day effects on the physiological responses to exercise in horses can have important implications on the planning of training schedules, working habits and competitions. The aim of the present study was to investigate the possible responses of some ocular physiological variables (OT and IOP) to physical exercise in show jumping horses during the morning (a.m.) and afternoon (p.m.) periods in correlation with rectal temperature.

## 2. Materials and Methods

Fourteen 10–15 year-old Italian Saddle Horses, with an average body weight of 520 ± 30 kg, were enrolled in the experimental design with their owners’ consent. Prior to the study, each animal underwent a complete clinical and ophthalmic exam and hematological and hematochemical analyses to determine its health status. Horses were housed in individual boxes (3.5 × 3.5 m) under a natural photoperiod (sunrise at 05:00; sunset at 21:00). All horses were fed three times a day (06:00, 12:00 and 20:00) with a diet based on good-quality hay and a mixed cereal concentrate, whereas water was available ad libitum. Environmental parameters were monitored during the experimental period using a multiparametric probe (Testo 400 Testo SE & Co. KGaA, Titisee-Neustadt, Germany), as micro-climatic conditions (ambient temperature, relative humidity and ventilation) may influence infrared thermography [27].

### 2.1. Exercise Protocol

All horses were trained from 9:00 to 10:00 a.m. and from 19:00 to 20:00 p.m., following a specific training program involving a 1 h session, including a walk (5′), trot (30′) and canter/gallop (20′); a jumping exercise (1′) was carried out on a 300 m long trail with eight jumps that were 90 cm high (4 vertical jumps, 3 spread fences and one double vertical and long jump), and at the end of the training program a few minutes of walking was included. Measurements were collected before and after the exercise during the morning (a.m.) and afternoon (p.m.) training sections. The hours of the day for the a.m. and p.m. sessions were chosen based on the circadian patterns of body temperature and IOP in horses [13,14,15,28], with minimal disturbance to the normal routine of the animal.

### 2.2. Measurements Assessment and Infrared Thermography

Each experimental recording was performed inside a specifically designed box for measurements with standardized environmental parameters. The same technician and veterinary performed all tests.

Thermal measurements of ocular temperature (OT) were taken using a SKM Italia S.r.l. FLIR T440 Camera (thermal accuracy of ±2% of the reading or 2 °C). Measurements were taken perpendicular to the eye, from the lacrimal caruncule, as this anatomical area is considered to be stress-sensitive for the animal. The FLIR 440T camera was positioned at a distance of 1.00 ± 0.01 m from each subject. The camera was handheld, to better follow all the animal’s head movements. The camera captured images in both the IR (7.5–13) nm 127 and the visible (0.4–0.7) mm range. Infrared radiation emitted by a surface closely depends on such micro-climatic conditions.

Intraocular pressure (IOP) was measured bilaterally using a Tono-Pen Applanation Tonometer (Tono-Pen XL, Mentor Ophthalmic, Reichert, Depew, NY 14043, USA) after a local anesthetic, oxybuprocaine hydrochloride (Novesina 0.4%, Novartis). The Tono-Pen was settled on the cornea, and a Sanitized Ocu-Film tip cover was used to minimize the risk of cross-contamination. Mean values were calculated. Measurements were repeated until the instrument percent error was <5% and three measurements were obtained at each pressure.

Rectal temperature (RT) was measured using a digital thermometer (model HI92704, Hanna Instruments), with resolution of 0.1 °C and a thermal accuracy of 0.3% for each reading, and this was inserted 15 cm into the rectum for each horse.

### 2.3. Statistical Analysis

Student’s *t*-test was used to evaluate statistical differences between the left and right eye. Two-way repeated-measures analysis of variance (ANOVA) was applied to evaluate the statistically significant effect of time (before vs. after) and of experimental conditions (a.m. vs. p.m.) on OT, IOP and RT. The Bland–Altman (B-A) test was performed to assess the agreement of RT and OT. In this test, the difference between the two techniques was plotted against their mean and the calculated limits of agreement.

The correlation coefficients (r) between RT and OT, between OT and IOP and between RT and IOP were determined. Regression lines and 95% confidence intervals for the different data recorded under each experimental condition were determined. Differences were considered statistically significant for *p* = 0.05. Statistical analysis was performed by using Graph Pad Prism 8 software (v8.4.2).

## 3. Results

Environmental conditions (ambient temperature, relative humidity and ventilation) were monitored during the experiment; they have been expressed as average values for a.m. and p.m. (ambient temperature: 18 °C–22 °C; relative humidity: 50–70%; ventilation: 0–1.5 m/s).

**Figure 1:**
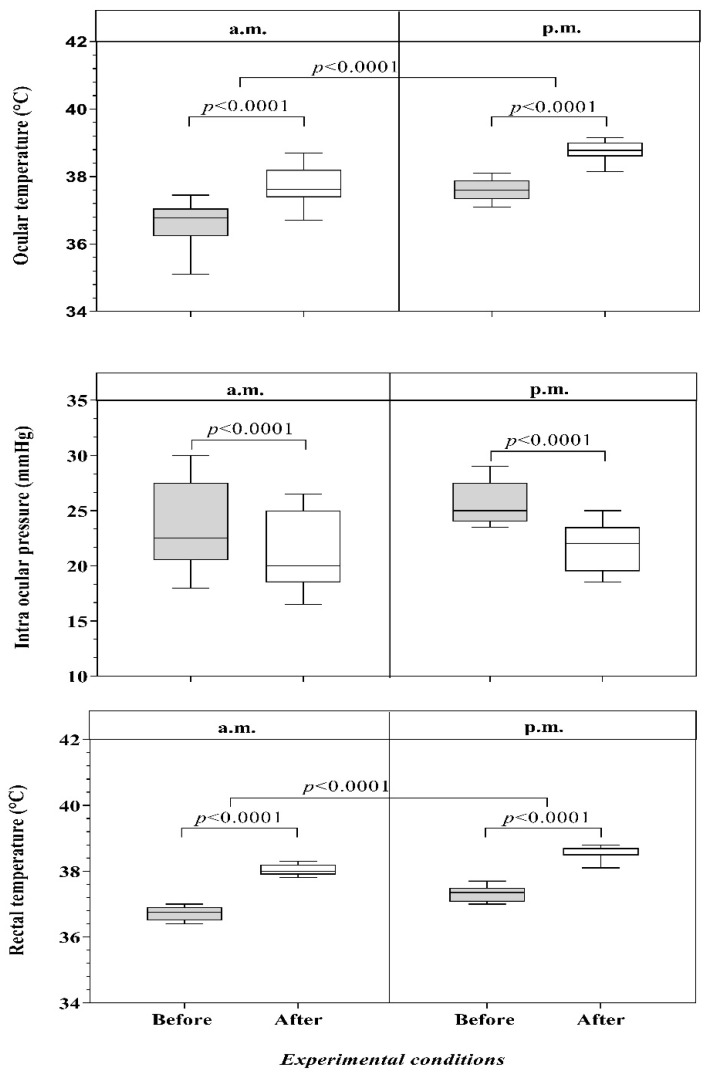
Comparison of ocular temperature, intraocular pressure and rectal temperature measured in the a.m. and p.m. hours before (grey boxes) and after exercise (white boxes) in 14 horses. Statistical significance: *p* < 0.0001.

The application of Student’s *t*-test showed no significant differences between the left and the right eye (*p* > 0.05) in terms of OT and IOP values; therefore, the mean values of the two eyes were considered in the following statistical analyses. Our data are expressed as mean ± standard deviation (SD) as showed in Table 1. Our results are reported in Figure 1. Two-way repeated-measures ANOVA showed a significant effect of time (before vs. after) on OT, IOP and RT (*p* = 0.0001). A statistically significant effect of experimental conditions (a.m. vs. p.m.) on OT and RT (*p* = 0.0001) was observed. No significant effect of experimental conditions on IOP was found (*p* = 0.0726).

**Figure 2:**
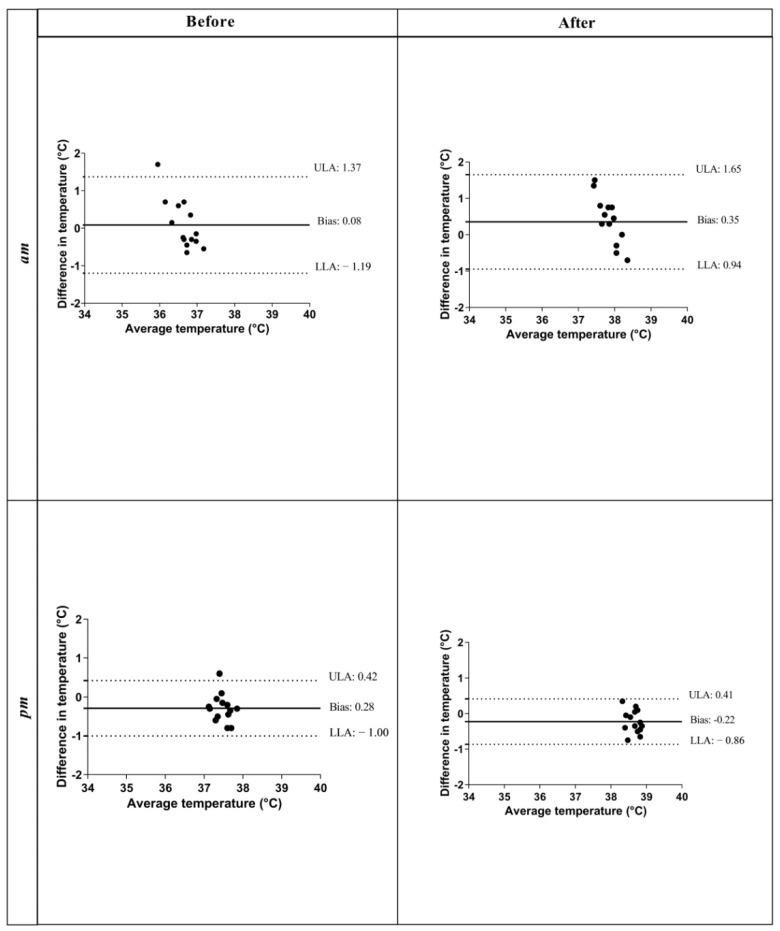
Bland–Altman plots of ocular temperature and rectal temperature, measured during a.m. hours before and after exercise, and during p.m. hours before and after the exercise. Average temperatures were plotted against differences with temperatures. Dotted lines represent upper and lower limits of agreement (ULAs and LLAs). The solid black line represents the mean difference or the bias.

We observed agreement in the results between RT and OT, as shown showed in B-A plots at each time point of the monitoring period (a.m. before exercise; a.m. after exercise; p.m. before exercise and p.m. after exercise). Particularly, an agreement between the values of RT and OT was found as the data fell within the upper and lower limits of agreement, as shown in Figure 2. The B-A test was used to plot the agreement in ocular and rectal temperatures. For OT and RT recorded during a.m. hours before exercise, the greater bias was 0.08 °C, with limits of agreement (LOAs) showing that the discrepancy between OT and RT for an individual animal ranged from −1.19 to 1.37. For OT and RT recorded during the a.m. hours after exercise, the bias was 0.35 °C, with LOAs ranging from 0.94 to 1.65; on p.m. hours before exercise, the bias was −0.28 °C, with LOAs ranging from −1.00 to 0.42 and for p.m. hours after exercise the bias was −0.22 °C, with LOAs ranging from −0.86 to 0.41.

**Figure 3:**
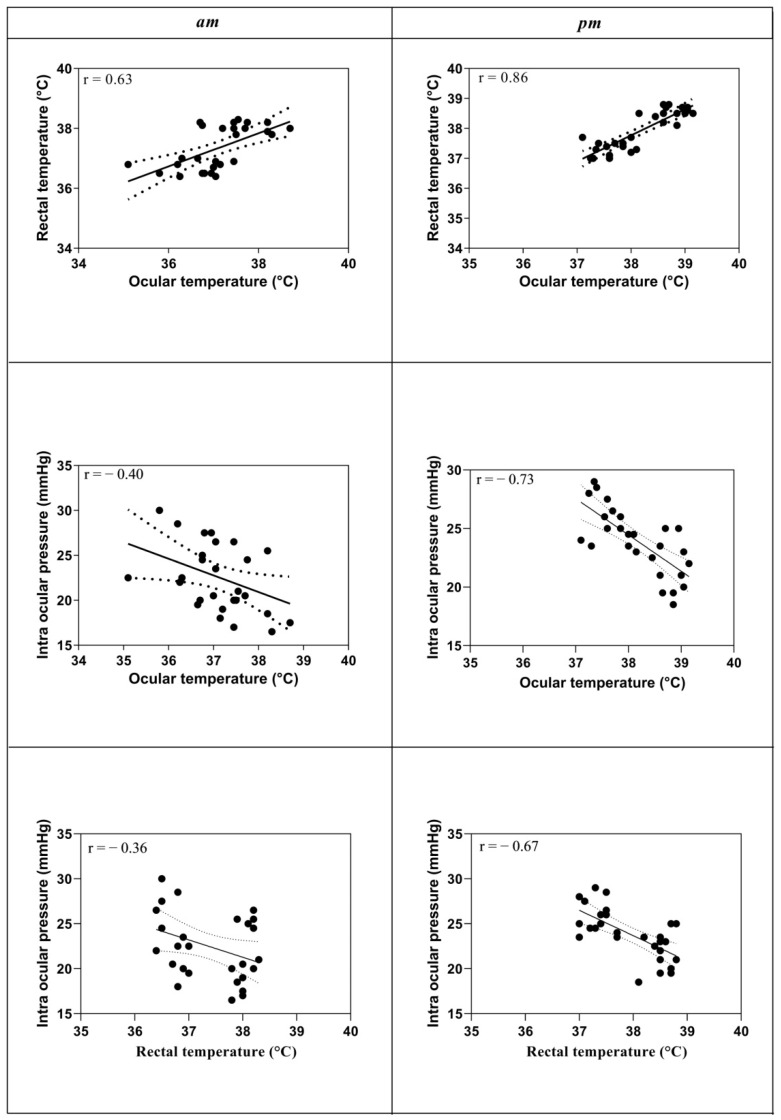
Linear regression values between ocular temperature and rectal temperature during the a.m. hours before and after exercise and between ocular temperature and rectal temperature during the p.m. hours before and after exercise. Linear regression analysis between ocular temperature and intraocular temperature during the a.m. hours before and after exercise and during the p.m. hours before and after exercise. Linear regression analysis between rectal temperature and intraocular temperature during the a.m. hours before and after exercise and during the p.m. hours before and after exercise.

The mean OT was positively correlated with RT in the a.m. hours (*p* = 0.0003; r = 0.63) and in the p.m. hours (*p* = 0.0001; r = 0.86). IOP showed a significant negative correlation with OT in the a.m. hours (*p* = 0.04; r = −0.40) and in the p.m. hours (*p* = 0.0001; r = −0.73). IOP showed a significant negative correlation with RT in the a.m. hours (*p* = 0.05; r = −0.36) and in the p.m. hours (*p* = 0.0001; r = −0.67), as shown in Figure 3.

## 4. Discussion

Many metabolic and physiological changes may occur in the body during exercise [24,29]. The comparison between the left and right eyes showed no differences in terms of both the parameters recorded. IOP was within the physiological range indicated for horses [15].

In the literature, some contradictory results have been obtained regarding the increase or decrease in OT values in response to physical exercise. It is likely that differences in data collection methodologies between studies, such as the time of sampling, may account for these differences [30]. Infrared thermography is able to effectively measure OT changes during exercise, which may be related to stress levels in show-jumping horses during daily training [2].

Our results showed a significant increase in OT and RT after exercise both in the a.m. and p.m. hours, as reported for humans in response to stress, mediated by the sympathetic nervous system [31]. In accordance with other studies, body temperature increased after exercise as a result of muscle metabolism activation, primarily due to skeletal muscle contraction, and the consequently increasing blood flow in various regions of the body to improve oxygen supply and heat dissipation [32].

Therefore, our results demonstrated an association between increased body temperatures and IOP variations during exercise [33]. OT has been observed to be significantly related to rectal temperature, confirming that both measurements may be carried out to detect hyperthermic changes during physical exercise in horses [27,34].

Based on the significant correlation between OT and RT found in this study, it can be assumed that ocular temperature behaves in a similar manner to rectal temperature. Both core and surface temperature of the body can reveal how animals cope with the surrounding environmental changes. Animals’ adaptation mechanisms to changes in environmental conditions consist of the activation of the HPA axis, causing the redistribution of blood and possibly promoting dilation of the blood vessels of the periocular musculature, with a consequent increase in body temperature, which has led to its association with the body’s response following acute stress of short duration [30,32].

Our findings suggest that OT may be considered as an alternative tool for non-invasive central temperature measurement as an indicator of horses’ physical fitness after a day of work. The superficial areas of the eye are rich in capillary beds, innervated by the sympathetic system, thus representing an ideal place to measure local changes in blood flow as a consequence of the activation of the autonomic nervous system [1,6,12,30,35,36].

The use of a thermo-camera allows for the rapid recording of the ocular temperature, compared to the digital thermometer used for measurements of rectal temperature. The results of this study support the use of IRT as an additional, rapid and non-invasive method for measuring body temperature [11].

As shown in Figure 1, both temperatures showed higher values before and after exercise during the p.m. training section than during the a.m. training section. The increase observed in the p.m. period resulted from the endogenous circadian pattern of body temperature. In horses, as diurnal animals, the increased heat production resulting from severe muscular work in the morning can elevate the body temperature to the usual p.m. values; the same exercise in the evening hours, on the other hand, can increase the risk of hyperthermia. In this regard, an excessive increase in body temperature has been found to reduce exercise performance. The studied parameters (IOP and RT) were observed to show a circadian rhythm that exhibited the presence of acrophase during the nocturnal period for RT and at the end of the photoperiod for IOP; therefore, we decided to conduct both a.m. and p.m. training sessions to avoid subjecting the animals to circadian stress monitoring [14,37].

Furthermore, our results showed a significant statistical decrease in IOP after exercise, in contrast to other findings [3,23]. No statistical effect was found on IOP between the a.m. and p.m. hours, suggesting that IOP is not affected by changes in the thermoregulation system which is activated between morning and afternoon as the body temperature increases after exercise [3,38]. The decrease in IOP observed after physical exercise may be associated with cardiovascular, systemic and hemodynamic adaptations intra- and post-exercise (such as changes in the heart rate, elevated systolic and diastolic blood pressure), which may be necessary to ensure the proper supply of oxygen and blood-transported substrates to activate the muscular system during exercise, along with the release of metabolites [22,24,29].

The degree of IOP reduction varies from study to study as a result of different experimental methods, intensities and durations of the workload [3,22,24,25,29]. The intensity of the workload was observed to influence hematological indicators useful in the assessment of the fitness of athletic horses, as well as the aerobic working capacity, the hydration status and the electrolyte balance [39,40].

In accordance with these observations, the decrease in IOP observed after exercise may be correlated with an increased serum osmolarity, associated with dehydration status and an increase in osmotic components. The osmotic disequilibrium between serum and aqueous humor may shift water from the aqueous and vitreous humor into the serum, triggering ocular dehydration, exhibited by the IOP reduction [29]. The present study has some limitations, such as the small sample size used. Although the small sample size may be warranted by the narrow inclusion criteria necessary to obtain a homogeneous sample of healthy animals, further studies are needed on a larger number of horses to confirm our results in different environmental conditions.

## 5. Conclusions

We can conclude that exercise leads an increase in OT, probably determined by physiological changes caused by exercise, as is the case for the rectal temperature. Such changes, promoting peripheral vasodilatation, likely affect IOP. During exercise, both temperature and pressure change; therefore, our results indicated a significant reduction in IOP after exercise, and it would be interesting to consider IOP and OT as indicators to determine (in a simple and noninvasive way) the physiologic status of horses during physical exercise [39]. Furthermore, we suggest the use of OT as a good replacement for other methods used to monitor body temperature in all cases in which the recording of rectal temperature is not possible. Future studies should be suggested in order to standardize the workload and the different physical effort intensities that may influence horses’ physical fitness after exercise and athletic horses’ welfare during sporting competitions.

## Figures and Tables

**Table 1:** Mean values ± SD of ocular temperature, intraocular pressure and rectal temperature, recorded before and after exercise during the a.m. and p.m. hours, together with their statistical significances. Capital letters (A vs. After; B vs. p.m. hours) indicate statistically differences between times and between experimental conditions.

**Parameters**	**a.m.**	
	**before**	**after**
*Ocular temperature (°C)*	36.61 ± 0.62 ^AB^	37.69 ± 0.58
*Intraocular pressure (mm/Hg)*	25 ± 3.18 ^A^	22.27 ± 3.16
*Rectal temperature (°C)*	36.69 ± 0.22 ^AB^	38.04 ± 0.16
**Parameters**	**p.m.**	
	**before**	**after**
*Ocular temperature (°C)*	37.62 ± 0.31 ^A^	38.76 ± 0.28
*Intraocular pressure (mm/Hg)*	26 ± 1.99 ^A^	22.32 ± 2.15
*Rectal temperature (°C)*	37.33 ± 0.24 ^A^	38.53 ± 0.20

## Data Availability

The datasets used and/or analyzed during the current study are available from the corresponding author on reasonable request.

## References

[1] Schaefer A.L., Matthewes L.R., Cook N.J., Webster J., Scott S.L. Novel noninvasive measures of animals welfare. Proceedings of the Animal Welfare and Behaviour: From Science to Solution, Joint NAWAC/ISAE Conference.

[2] Seabra J.C., Dittrich J.R., Martinez do Vale M.M., do Rocio J., de Hollanda R.S. (2019). Eye temperature change in response to race training in thoroughbred horses at the Jockey Club. Arch. Vet. Sci..

[3] Moura M.A., Rodrigues L.O.C., Waisberg Y., De Almeida H.G., Silami-Garcia E. (2002). Effect of submaximal exercise with water ingestion on intraocular pressure in healthy humans males. Braz. J. Med. Biol. Res..

[4] Salak-Johnson J.L., McGlone J.J. (2007). Making sense of apparently conflicting data: Stress and immunity in swine and cattle. J. Anim. Sci..

[5] Giannetto C., Aragona F., Arfuso F., Piccione G., De Caro S., Fazio F. (2022). Diurnal variation in rectal and cutaneous temperatures of horses housed under different management conditions. Int. J. Biometeorol..

[6] Dunbar M.R., Johnson S.R., Rhyan J.C., Mccollum M. (2009). Use of infrared thermography to detect thermographic changes in mule deer (*Odocoileus hemionus*) experimentally infected with foot-and-mouth disease. J. Zoo Wildl. Med..

[7] Stewart M., Webster J.R., Schaefer A.L., Cook N.J., Scott S.L. (2005). Infrared thermography as a non-invasive tool to study animal welfare. Anim. Welf..

[8] Valera M., Bartolomé E., Sánchez M.J., Molina A., Cook N., Schaefer A.L. (2012). Changes in eye temperature and stress assessment in horses during show jumping competitions. J. Equine Vet. Sci..

[9] McGreevy P., Warren-Smith A., Guisard Y. (2012). The effect of double bridles and jaw-clamping crank nosebands on temperature of eyes and facial skin of horses. J. Vet. Behav. Clin. Appl. Res..

[10] Bartolomé E., Sánchez M.J., Molina A., Schaefer A.L., Cervantes I., Valera M. (2013). Using eye temperature and heart rate for stress assessment in young horses competing in jumping competitions and its possible influence on sport performance. Animal.

[11] De Mira M.C., Lamy E., Santos R., Williams J., Vaz Pinto M., Martins P.S., Rodrigues P., Marlin D.L. (2021). Salivary cortisol and eye temperature changes during endurance competitions. BMC Vet. Res..

[12] Johnson S.R., Hussey S.B., Morley P.S., Traub-Dargatz J.L. (2011). Thermography eye temperature as an index to body temperature in ponies. J. Equine Vet. Sci..

[13] Giannetto C., Piccione G., Giudice E. (2009). Daytime profile of the intraocular pressure and tear production in normal dog. Vet. Ophthal..

[14] Giannetto C., Assenza A., Fazio F., Casella S., Piccione G. (2009). Circadian intraocular pressure and tear production profile in horses. Arch. Vet. Ital..

[15] Piccione G., Giannetto C., Fazio F., Giudice E. (2010). Influence of different artificial lighting regimes on intraocular pressure circadian profile in the dog (*Canis familiaris*). Exp. Anim..

[16] Harada Y., Naoi N. (2004). Corneal elasticity as a measure of intra-ocular pressure: A controlled clinical examination. KOBE J. Med. Sci..

[17] Wada S. (2006). Changes of intraocular pressure uveitic horses. J. Equine Vet. Sci..

[18] Hendrix D.V.H. (2007). Diseases and surgery of the canine anterior uvea. Essentials of Veterinary Ophthalmology.

[19] Del Sole M.J., Sande P.H., Bernardes J.M., Aba M.A., Rosenstein R.E. (2007). Circadian rhythm of intraocular pressure in cats. Vet. Ophthalmol..

[20] Gelatt K.N., Brooks D.E., Käberg M.E. (2007). The canine glaucomas. Essentials of Veterinary Ophthalmology.

[21] Martin B., Harris A., Hammel T., Malinovsky V. (1999). Mechanism of exercise-induced ocular hypotension. Investig. Ophthalmol. Vis. Sci..

[22] Qureshi I.A., Xi X.R., Mbbs Huang Y.B., Sc B., Wu X.D. (1996). Magnitude of decrease in intraocular pressure depends upon intensity of exercise. Korean J. Ophtalmol..

[23] Dickerman R.D., Smith G.H., Langham-Roof L., McConathy W.J., East J.W., Smith A.B. (1999). Intra-ocular pressure changes during maximal isometric contraction: Does this reflect intra-cranial pressure or retinal venous pressure?. Neurol. Res..

[24] Giudice E., Giannetto C., Casella S., Piccione G. (2010). The effect of aerobic exercise on intraocular pressure in horses. Acta Vet. Brno.

[25] Harris A., Malinovsky V., Martin B. (1994). Correlates of acute exercise-induced ocular hypotension. Investig. Ophthalmol. Vis. Sci..

[26] Wylęgała A. (2016). The effect of physical exercises on ocular physiology: A review. J. Glaucoma.

[27] Giannetto C., Di Pietro S., Falcone A., Pennisi M., Giudice E., Piccione G., Acri G. (2021). Thermographic ocular temperature correlated with rectal temperature in cats. J. Ther. Biol..

[28] Piccione G., Caola G., Refinetti R. (2002). Maturation of the daily body temperature rhythm in sheep and horse. J. Therm. Biol..

[29] Ashkenazi I., Melamed S., Blumenthal M. (1992). The effect of continuous strenuous exercise on intraocular pressure. Investig. Ophthalmol. Vis. Sci..

[30] Arfuso F., Acri G., Piccione G., Sansotta C., Fazio F., Giudice E., Giannetto C. (2022). Eye surface infrared thermography usefulness as a noninvasive method of measuring stress response in sheep during shearing: Correlations with serum cortisol and rectal temperature values. Physiol. Behav..

[31] Levine J.A., Pavladis I., Cooper M. (2001). The face of fear. Lancet.

[32] Trindade P.H.E., de Camargo Ferraz G., Pereira Lima M.L., Negrão J.A., Paranhos da Costa M.J.R. (2019). Eye surface temperature as a potential indicator of physical fitness in ranch horses. J. Equine Vet. Sci..

[33] Piccione G., Giannetto C., Marafioti S., Casella S., Assenza A., Fazio F. (2011). Comparison of daily rhythm of rectal and auricular temperatures in horses kept under a natural photoperiod and constant darkness. J. Therm. Biol..

[34] Zanghi B.M. (2016). Eye and ear temperature using infrared thermography are related to rectal temperature in dogs at rest or with exercise. Front. Vet. Sci..

[35] Eddy A., Van Hoogmoed L., Snyder J. (2001). The role of thermography in the management of equine lameness. Vet. J..

[36] Hodgson D.R., McGowan C.M., McKeever K.H. (2014). The Athletic Horse: Principles and Practice of Equine Sports Medicine.

[37] Piccione G., Giannetto C., Fazio F., Giudice E. (2009). Daily rhythm of tear production in normal dog maintained under different light/dark cycles. Res. Vet. Sci..

[38] Gerardi B., Denadai D.S., Pereira M.S., Chaves A.A., Barbosa J.P.B., Peiró J.R., Feitosa F.L.F., Mendes L.C.N. (2019). Use of infrared thermography in Quarter Horse submitted to team roping. Pesqui. Vet. Bras..

[39] Piccione G., Giannetto C., Fazio F., Di Mauro S., Caola G. (2007). Haematological response to different workload in jumper horse. Bulg. J. Vet. Med..

[40] Piccione G., Giannetto C., Assenza A., Fazio F., Caola G. (2007). Serum electrolyte and protein modification during different workload in jumper horse. Comp. Clin. Pathol..

